# Egg introduction during complementary feeding according to allergic risk: not just for peanuts!

**DOI:** 10.1186/s13052-018-0521-x

**Published:** 2018-07-06

**Authors:** Elvira Verduci, Annamaria Bianchi, Marta Brambilla, Mauro Calvani

**Affiliations:** 1 Department of Pediatrics, San Paolo Hospital, Milan, Italy; 2 Department of Health Science, University of Milan, Milan, Italy; 3 Department of Pediatrics, S. Camillo-Forlanini Hospital, Rome, Italy

**Keywords:** Complementary food, Allergy prevention, Egg allergy

## Abstract

The relationship between the timing of introduction of complementary foods and later allergy is a topic of current discussion. Although the European Society for Pediatric Gastroenterology Hepatology and Nutrition (ESPGHAN) has recently recommended that potentially allergenic foods may be introduced when complementary feeding is commenced, any time after 4 months, recommendations about egg introduction would be needed mainly for infants with high risk of developing food allergy. Before the first administration in these infants an adequate topical therapy and an evaluation of whole egg–specific IgE serum antibody levels or skin prick tests for egg should be recommended.

To the Editor,

Early regular oral egg exposure in infants with eczema prevent later egg allergy. However caution needs to be taken at the first ingestion because many of them have sensitization already by 4 months of age and showed severe reaction.

The term “complementary foods” includes all solid and liquid foods other than breast milk or infant formula. Complementary foods are necessary for both nutritional and developmental reasons and are an important stage in the transition from milk feeding to family foods [1]. There is an important and controversial relationship between the timing of the introduction of complementary foods and later food allergies. Regarding the exposure to potentially allergenic foods, including cows’ milk, egg, fish, gluten, peanut, and seeds, international recommendations suggest that there is an increased risk of allergy if solids are introduced before 3 to 4 months, but there is no evidence that delaying the introduction of allergenic foods beyond 4 months reduces the risk of allergy, either for infants in the general population or for those with a family history of atopy [2]. Thus, potentially allergenic foods may be introduced when complementary feeding is commenced any time after 4 months (17 weeks beginning at the 5th month of life), both in breast-fed and formula-fed infants and independently from the risk of atopy, according to the recent updated ESPGHAN recommendation on complementary feeding [1]. Also exclusive or full breast-feeding should be promoted for at least 4 months (17 weeks) and exclusive or predominant breast-feeding for approximately 6 months is considered a desirable goal [1].

In the last decade, prospective interventions have and are studying the hypothesis that the early introduction of potentially allergenic foods can prevent the development of food allergy in the general population or in children with a risk of developing food allergy (atopic dermatitis, atopic familiarity, food sensitization). While for peanuts, a guidance on the timing of the introduction of peanuts stratifying the child’s population by the risk of developing allergy has been provided [3], recommendations about egg introduction are lacking in infants at high allergic risk where recommendations would be needed, especially in those countries where the prevalence of egg allergy is certainly greater than that of peanuts. Where the prevention of egg allergy is a concern, the results of 5 interventional trials have been published (STAR [4], STEP [5], HEAP [6], BEAT [7], PETIT [8]) in the last two years. A recent systematic review and meta-analysis [9] concluded that there was moderate evidence from these 5 trials (1915 participants) that egg introduction at 4 to 6 months was associated with reduced egg allergy risk (risk ratio [RR] 0.56 [95% CI 0.36 – 0.87], I^2^ = 36%, *P* = 0.009). However, these systematic review conclusions should be evaluated in the context of limitations in the primary studies, and of the heterogeneity of the included studies. The imprecise effect estimates, issues regarding indirectness, and inconclusive trial sequential analysis findings all need to be taken into account, together with a careful assessment of the safety and acceptability of early egg introduction in different populations. Heterogeneity for egg introduction was due mainly to PETIT study [9]. Indeed in this study small incremental doses of heated egg powder have been administered at 6 to 12 months in infants with atopic dermatitis in association with an aggressive dermatitis control therapy. This approach could justify the interesting results obtained from this study that could have influenced the conclusion of the systematic review. Moreover, studies showed that early introduction at 4-6 months of hen’s egg could lead to a large number of allergic reactions, even within the general population. Indeed, four of the trials [4–7] reported allergic reactions (possible even severe) at the ingestion of raw pasteurized egg, due to prior sensitization, ranging from 4.7% of infants with atopic heredity and skin prick test (SPT) to egg white < 2 mm [7], to 31% of the infants affected by moderate-severe atopic dermatitis [4]. Otherwise in the PETIT study no infant withdrew because of adverse reactions caused by the trial powder, even though this study enrolled high-risk infants with atopic dermatitis. However, in this trial, a very low daily dose of heated egg powder was introduced orally at 6 months (25 mg of egg protein which is equivalent to 0.2 g of egg). Therefore the quantity of allergen and the degree of cooking seem to influence the probability of an allergic reaction, even at the first ingestions of egg.

From a practical point of view, the dose administered in the PETIT study is not easy to replicate given that egg is not commonly eaten in powder form. Indeed, considering that a medium sized egg weighs about 60 g, and that the edible part is about 50 g, a 1/250th of an egg should be administered to give 0.2 g of egg (or 25 mg of protein)!

In conclusion, while waiting for other studies to clarify the preventive effect and the safety of an early introduction of heated egg, some indications about egg introduction during complementary feeding according to allergic risk can be provided based on existing recommendations for other highly allergenic nutrients. When the introduction of egg to infants at very high-risk (with moderate-severe atopic dermatitis) is considered, it seems reasonable to adopt a behavior similar to that proposed for peanuts (Fig. 1). In these infants an adequate topical therapy should be recommended, as well as evaluation of whole egg–specific IgE serum antibody levels or performing skin prick tests for egg before the first administration. Other tests, e.g. patch test, are not useful [10]. If skin prick test or sIgE are negative, cooked egg can be introduced at low quantity when complementary feeding is commenced. This, because in two trials that enrolled un-sensitized infants, with egg white extract SPT < 2 mm [6] or hen’s egg sIgE < 0.35 kU_A_/L [7] the introduction of raw hen’s egg (un-cooked) at between 4 and 6 months, induced mild or moderate reactions. If skin prick test or sIgE are positive, egg must be introduced in a specialized setting with emergency support immediately available and under the supervision of an allergist with expertise in this field.

**Fig. 1:**
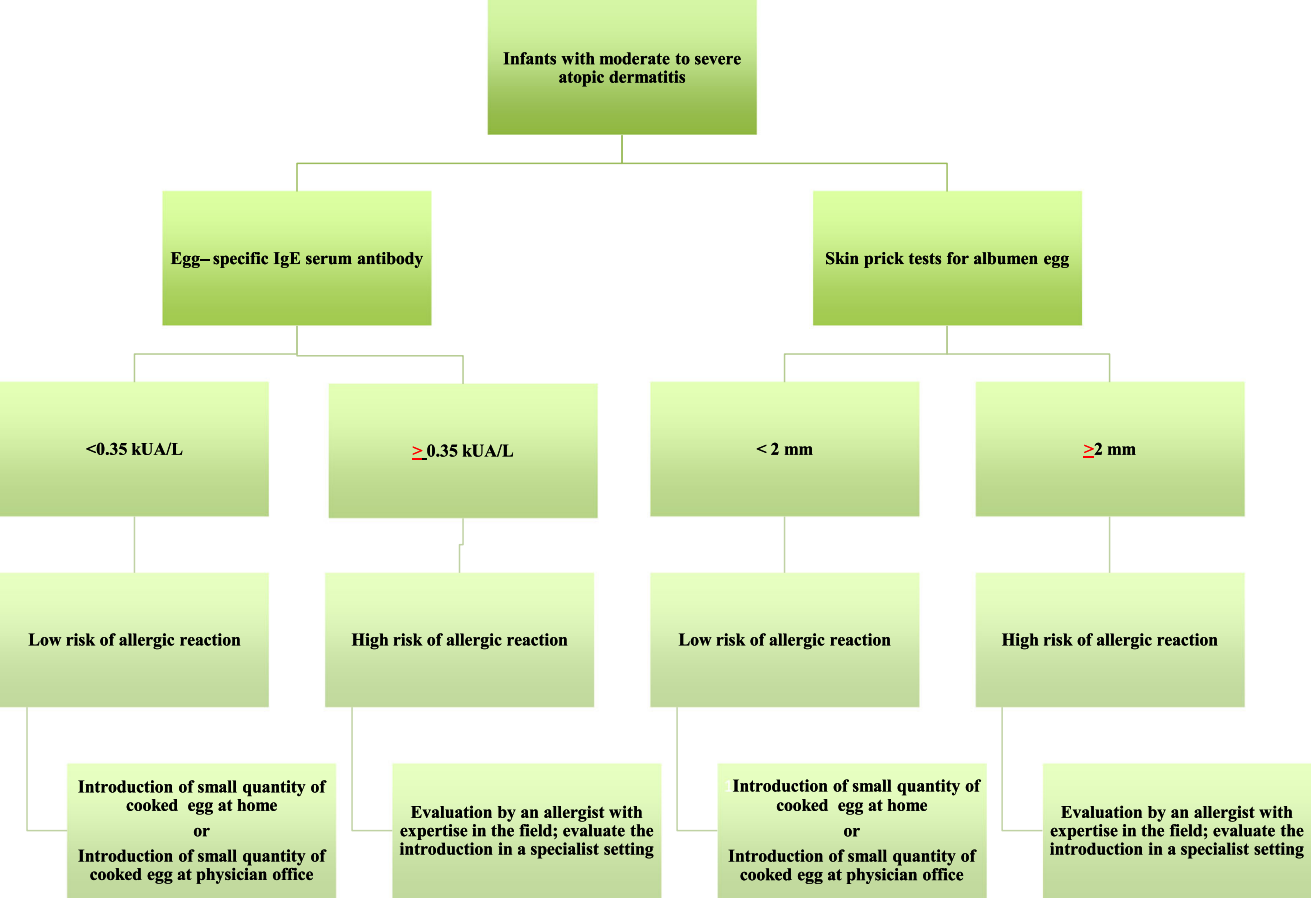
Egg introduction in infant with atopic dermatitis: a proposal of algorithm

## Abbreviation

SPT: skin prick test
